# On the Influence of Corrosion on the Load-Carrying Capacity of Old Riveted Bridges

**DOI:** 10.3390/ma13030717

**Published:** 2020-02-05

**Authors:** Jozef Gocál, Jaroslav Odrobiňák

**Affiliations:** Department of Structures and Bridges, Faculty of Civil Engineering, University of Žilina, Univerzitná 8215/1, 010 26 Žilina, Slovakia; jozef.gocal@uniza.sk

**Keywords:** corrosion of steel, riveted bridges, degradation, load-carrying capacity

## Abstract

Steel corrosion is one of the most dominant factors in the degradation of transport infrastructure. This article deals with the impact of the atmospheric corrosion of structural steel on the load-carrying capacity of old riveted bridge structures. A study on the impact of corrosion losses on the resistance and, thus, the load-carrying capacity of eight chosen bridge members with riveted I-sections from three different bridge substructures is presented. The load-carrying capacity calculation is carried out using modern procedures and on the basis of the diagnosed state of the structural elements. Within the analysis of the results, the need for long-term in situ corrosion measurements, as well as the need for regular inspections on the existing bridges are also discussed.

## 1. Introduction

### 1.1. Degradation of Bridges due to Corrosion

Existing bridges are structures that reflect not only the level of the society in which they were built, but also the cultural and economic power of the present generations, as they reflect the care for these inherited engineering works. Therefore, professionals centered on bridges must be consistent in all activities related to the design, construction, and management of bridges from the initial idea to the end of their service life. Despite efforts during the design and construction of bridge structures, various damages and failures occur during their exploitation. Initially, small defects can gradually develop into failures significantly affecting load capacity and traffic safety.

Today, transport, energy, and environmental infrastructure structures (including bridge structures) account for approximately 70% of national assets in European countries. Their operation, maintenance, repair, and reconstruction consume around 35% of the total material and energy consumption and produce about 30% of all environmental burdens and waste. The above information highlights the impact of transport infrastructure on the economies of the countries and environments in which we live. The public usually perceives this fact only marginally, until the underestimated inspections and maintenance activities result in fatal consequences, such as the known collapses of footbridges or bridges in our and surrounding countries. Unfortunately, only then, the public discussion, supported by the media environment, immediately focuses on the management and maintenance of bridge structures.

The most powerful tool for the evaluation of bridge structures is diagnostics-supported determination of the load-carrying capacity of bridges and estimation of their residual life. These are extremely demanding and responsible tasks, in which the maximum permissible traffic load of the bridge is calculated in reverse form from the existing condition. A common problem is how to do these analyses with respect to environmental degradation processes and their future development.

The main factors influencing the condition of bridges, besides natural changes of the material, are their hidden structural defects and increasing traffic intensities, especially the degradation processes taking place in structural elements, which are caused by environmental load from the surrounding environment [1]. Polluted air has a significant impact on the degradation of all materials used in the transport infrastructure and throughout the construction industry. As the share of human-caused pollution is increasing, the polluted air becomes considerably more aggressive with a greater impact on the structural parts of bridges and footbridges. The damages caused by corrosion and related environmental degradation phenomena annually account for about 3%–4% of GDP in developed countries [2]. Several studies estimate that from 25% to 30% of annual corrosion costs could be saved if optimum corrosion management practices were employed [3]. Sophisticated expert assessment of damages and failures of bridge structures in terms of their impact on bridge reliability is an important task in determining the bearing capacity of bridges and designing their reconstruction.

### 1.2. Influence of Corrosion on Load-Carrying Capacity

As mentioned above, the corrosion degradation of structural steel has a significant impact on the bridge’s reliability, in particular its safety and durability. Corrosion losses reduce the effective cross-sectional area of the load-bearing elements and thereby reduce their mechanical resistance to the effects of loads on the superstructure of the bridge. Depending on the level of safety of the individual load-bearing elements, it may happen during the service life of the bridge that the element reduced by progressive corrosion is no longer able to transmit the load effects, in particular the operational loads for which the bridge was primarily designed. The ability of the bridge structure to transmit the effects of the traffic load is quantified by the so-called “load-carrying capacity” (LCC), which is a basic quantification indicator for the evaluation of existing bridges. LCC represents a criterion that is valid not only for future planning, but is also used as the decision parameters for the evaluation of the passage of the actual railway service load. In recent years, several European-wide research projects have been completed [4] (such as, e.g., [5,6,7,8]). These projects have led to the development of guidelines that provide state-of-the-art methods for the safety assessment of existing bridges. Within the frame of this trend, the newest recommendation for the determination of the load-carrying capacity of metal railway bridges is being developed [9]. The newly elaborated guidelines for the determination of load-carrying capacity of railway bridges in the Slovak Republic [10] and Czech Republic [11] are also based on the latest knowledge combining the actual design codes and experiences from the area of evaluation.

Within transport infrastructure, there are relatively many bridge structures older than 50 years in Slovakia. For example, only in the case of railway bridges out of a total of 2300 bridge structures, up to 28% are over 75 years old, and almost one fifth of the bridges are even older than 100 years [12]. Thus, it is obvious that monitoring and consequently taking into account degradation due to environmental load is a very important factor in evaluating these structures and determining their load-bearing capacity. The LCC is generally defined as the ratio Z of the limiting effects of the vertical variable traffic load (in terms of the corresponding limit state) to the effects caused by the design load model in the member. This ratio represents the factor by which the multiplied effects of the load model (stresses, internal forces, deformations, etc.), in combination with other applied loads, cause the occurrence of corresponding limit states. In the case of railway bridges, this factor defines a multiple of the Load Model 71 (LM71) [13]; therefore, the ratio representing the LCC is referred to as Z_LM71_. More details regarding LCC estimation can be found in [14,15].

In the case of riveted cross-sections of the old bridge structures, it is necessary to carry out several types of assessments in order to find a decisive check, which leads to the load-carrying capacity of the element itself. The impact of substantial imperfections of elements and parts of the steel structure should be taken into account in the global bridge analysis itself. Thus, major defects due to corrosion, in particular the significant reduction of the cross-section by corrosion, are to be included in the global analysis of the structural behavior already. Of course, any significant corrosion loss must also be taken into account in the verification of the cross-sections and members of the bridge structure.

## 2. Study Description

### 2.1. Inputs for Analysis

To point out how corrosion losses can reduce the resistance of a bridge member and its load-carrying capacity, the following study was executed. LCC of members with typical riveted I-sections of three old real railway bridges in service were calculated. The measured corrosion losses were taken into account in the process of bridge analysis and cross-section verifications. The main girders of two bridges were made of plate girders, while the last bridge had truss girders with both chords curved. All three bridges had a typical open member deck.

**Figure 1:**
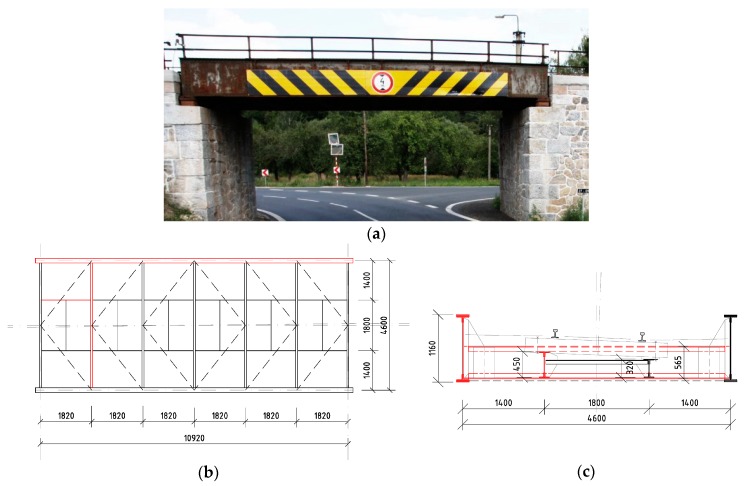
Bridge 1: the 142-year-old plate girder bridge with a bottom open member deck: (**a**) picture from the side; (**b**) schematic ground plan of the superstructure; (**c**) cross-section of the bridge; the chosen elements are drawn in red in (**b**) and (**c**).

The first bridge, designated as “Bridge 1”, is the smallest one, but it had been in the service for 142 years already. It bridges a railway line across a local road; thus, the span is only 10.92 m (Figure 1). For the presented study, the left stringer (outer side of the railway line directional curve), the second crossbeam, and the left (outer) main plate girders were chosen. These members emerged from the analysis as critical to the LCC of the bridge.

**Figure 2:**
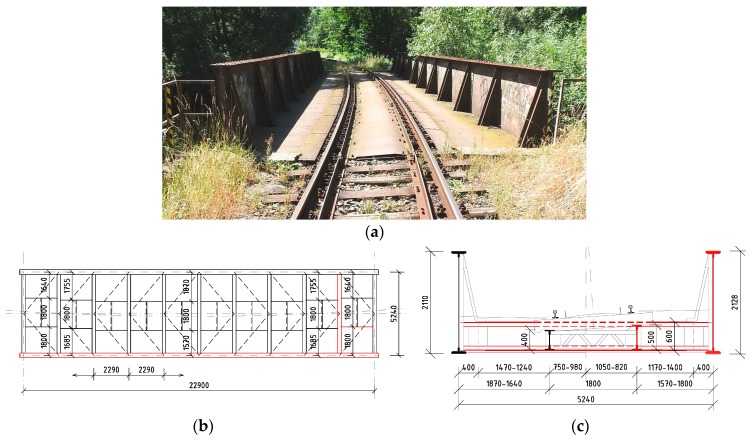
Bridge 2: the 109-year-old plate girder bridge with the bottom open member deck across a local stream: (**a**) picture of the bridge from the track; (**b**) schematic ground plan of the superstructure; (**c**) cross-section of the bridge; the chosen elements are drawn in red in (**b**) and (**c**).

As mentioned before, the second railway bridge is also the plate girder bridge with an open member deck (Figure 2). “Bridge 2” was built across local stream in 1910. The span of the main girders is 22.90 m, and their mutual axial distance is 5.24 m. For the load-carrying capacity of this structure, the determining members were: the last right (outer one) stringer, the ninth cross-beam, and the right main girder on the outer side of the railway line directional curve.

**Figure 3:**
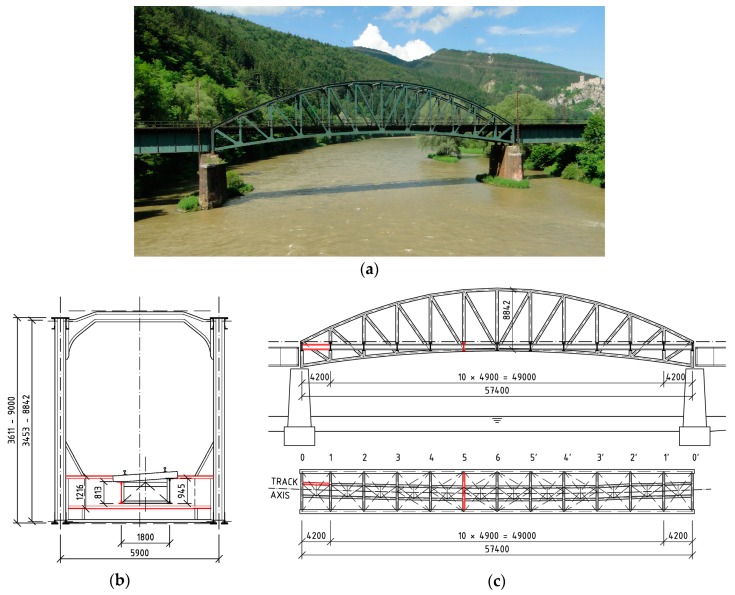
Bridge 3: the 76-year-old superstructure with the middle span built as the truss girder bridge with an intermediate open member deck: (**a**) picture from the side; (**b**) the cross-section; (**c**) schematic side view and ground plan of the superstructure; the chosen elements are drawn in red in (**b**) and (**c**).

The last bridge structure consisting of three simply supported superstructures was built on the main railway line. The main middle structure (“Bridge 3”) with a span of 57.4 m is 76 years old. It was built as truss girder bridge with an intermediate open member deck (Figure 3). In order to estimate the effect of corrosion on the resistance of the I-shaped cross-sections of this bridge, the first left (inner) stringer and the sixth crossbeam of the bridge deck were chosen, as they showed the lowest LCC.

The basic characteristic of each bridge and chosen member for the following study are summarized in Table 1.

First, detailed inspections of the condition and diagnostics were carried out on all three bridge structures. The necessary geometry and imperfection data were measured and verified. In situ measurements to determine the material characteristics were also performed. In the case of degradation caused by corrosion, the main focus was on the current corrosion attack.

**Figure 4:**
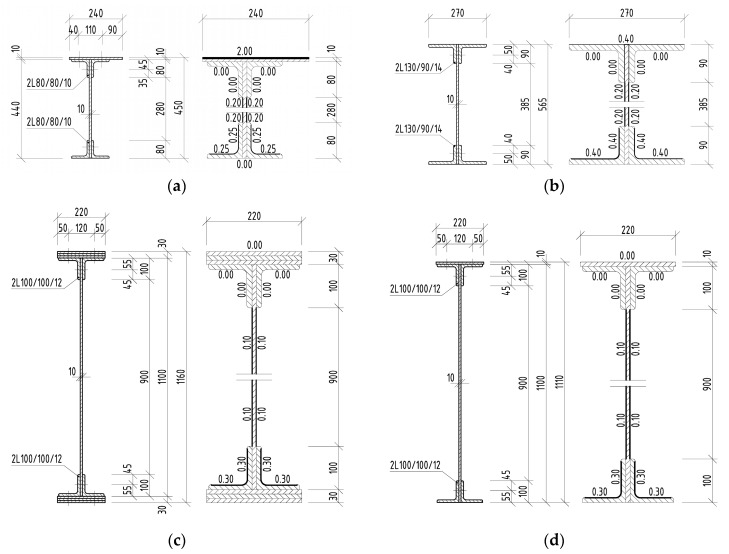
Riveted I-sections and average corrosion losses of elements in the case of Bridge 1: (**a**) left stringer; (**b**) crossbeam; (**c**) left main girder in the midspan; (**d**) left main girder in the support zone.

**Figure 5:**
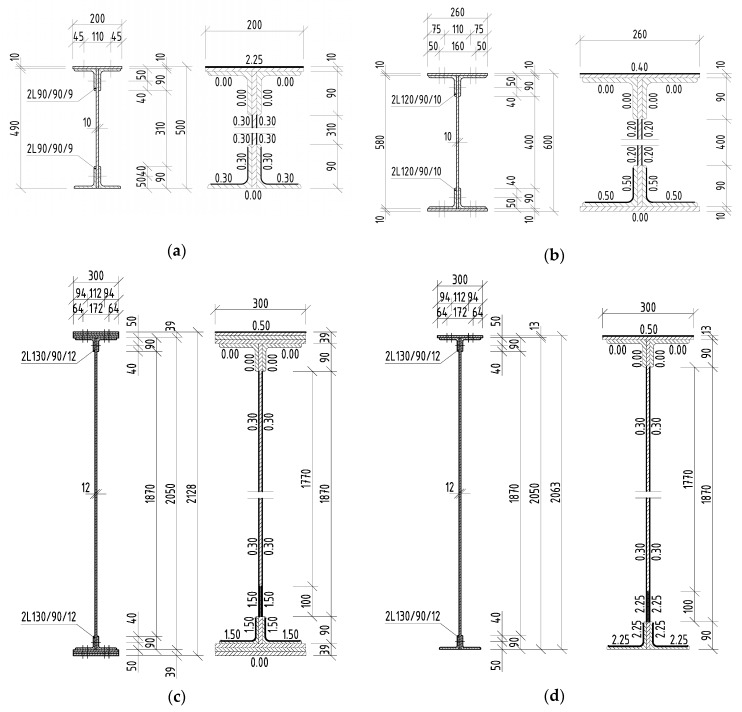
Riveted I-sections and average corrosion losses of elements in the case of Bridge 2: (**a**) right stringer; (**b**) crossbeam; (**c**) right main girder in the midspan; (**d**) right main girder near the support.

**Figure 6:**
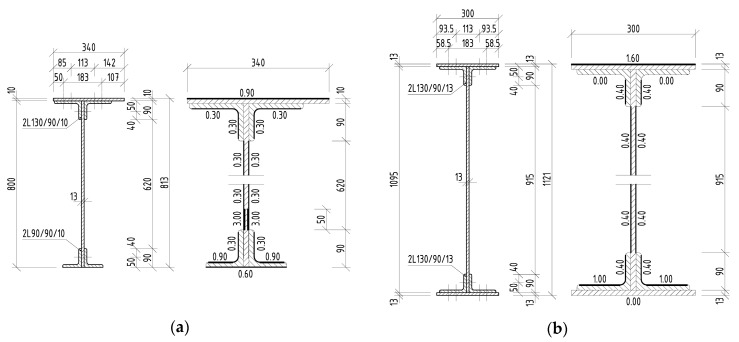
Riveted I-sections and average corrosion losses of deck elements in the case of Bridge 3: (**a**) first stringer; (**b**) crossbeam.

The in situ measurement of the corrosion attack of the elements took place most often after removal of the corrosion products using thickness gauges. Individual measured corrosion losses were recorded over the cross-section of the attacked element in several places. The number of measured places depended on the length of the element to be evaluated, the size of the cross-section of the element itself, as well as the cross-sectional structure and its corrosion damage. Subsequently, the data were statistically evaluated, and an effective cross-section of the riveted element was determined. In the case of the riveted I-sections listed above, the average corrosion losses are shown in Figure 4, Figure 5 and Figure 6, in which all dimensions are given in millimeters.

**Figure 7:**
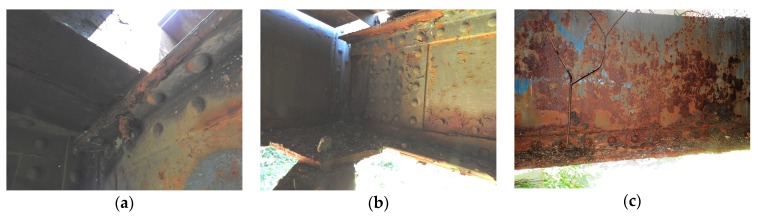
Examples of the degradation of the elements of Bridge 2: (**a**) local severe degradations of the top flange under the sleeper contact; (**b**) detail of the joint where the crossbeam, main girder, and bottom bracing are connected; (**c**) main girder; strong corrosion of the bottom flange and the bottom part of the girder web.

The photos in Figure 7 illustrate the conditions of some structural elements or the details of one of the bridges. Obviously, the corrosion attack was not always necessarily uniform and depended greatly on the position of a particular member or element in the structure, as well as on the position of the measured local point on the element itself.

### 2.2. Load-Carrying Capacity

The guideline [10] presents general rules and a methodology for determining the load-carrying capacity of the railway bridges. As corrosion can be described as a random process, the best way to analyze such an effect in time is through stochastic approaches [16]. Moreover, other inputs such as material properties and of course actions are also of a stochastic nature. If the more sophisticated calculation was the issue, stochastic numerical approaches replacing traditional finite element method (FEM) analysis are also available [17].

Anyway, the aim of the paper is to show the importance of corrosion losses in load-carrying capacity calculation. The worldwide-used semi-probabilistic method of load and resistance factor design (LRFD) was applied in this research. As the existing structures were to be analyzed, partial safety factors were calibrated. The methodology in guidance [10] for the modification of reliability indexes for the evaluation of existing bridges was taken into account. The basic concept of how the reliability levels were transformed into the design values of the material properties and load effects could also be found in the paper [15].

**Figure 8:**
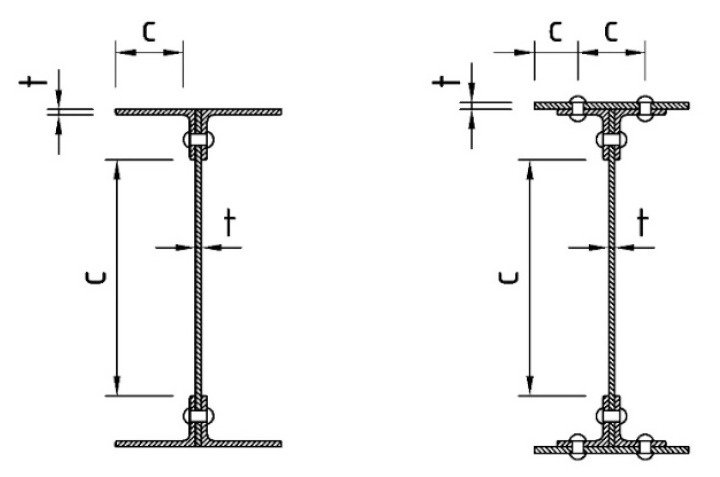
Definition of widths for the classification of riveted I-sections.

For the classification of the riveted cross-sections, the widths of the respective parts of the cross-section are defined in Figure 8. In contrast to the welded cross-sections, it was also necessary to verify the classification in terms of the distance of the rivets parallel to the direction of the applied stresses in addition to the transverse direction.

The determination of the LCC of cross-section under bending and tensile force or axial compression can be performed according to the Equation (1), in which the degradation due to corrosion is covered in cross-sectional parameters:

(1) $$Z_{LM71}=(1-\eta_{1,rs})/\eta_{1,LM71},$$

where:

(2) $$\eta_{1,rs}=N_{rs,Ed}/(A\times f_{yd})+M_{y,rs,Ed}/(W_{el,y}\times f_{yd})+M_{z,rs,Ed}/(W_{el,z}\times f_{yd}),$$

(3) $$\eta_{1,LM71}=N_{LM71,Ed}/(A\times f_{yd})+M_{y,LM71,Ed}/(W_{el,y}\times f_{yd})+M_{z,LM71,Ed}/(W_{el,z}\times f_{yd}),$$

and designations N_LM71,Ed_, M_y,LM71,Ed_, and M_z,LM71,Ed_ represent the design values of axial force and bending moments due to vertical variable rail traffic load effects including the dynamic factor, while N_rs,Ed_, M_y,rs,Ed_, and M_z,rs,Ed_ are the design, combination, or group values of axial force and bending moments due to other load effects acting simultaneously with the vertical rail traffic load. In the cross-sectional characteristics, A (the area), W_el,y_, and W_el,z_ (the section modules) of the riveted cross-section, the holes for rivets were excluded in the tensile area of the cross-section. Moreover, the cross-sectional characteristics took into account the degradation due to corrosion by varying the thickness of the respective cross-sectional part. Finally, the design value of steel yield stress could be obtained from f_yd_ = f_y_/γ_M0_, where γ_M0_ is a partial factor for the material and resistance of cross-sections.

The value of LLC obtained from (1) is valid if the shear force meets the condition:

(4) $$\eta_{3}=V_{Ed}/V_{Rd}=(Z_{\mathrm{LM}71}\times V_{\mathrm{LM}71,\mathrm{Ed}}+V_{\mathrm{rs},\mathrm{Ed}})/V_{Rd}\leq 0.5,$$

where V_LM71,Ed_ is the design value of shear force due to vertical variable rail traffic load effects represented by the LM71 including dynamic factors and V_rs,Ed_ is the design, combination, or group value of shear force due to other load effects acting simultaneously with the vertical rail traffic load. The minimum from the design values of the shear resistance of the cross-section or design value of shear resistance of the web is designated as V_Rd_.

If the above-mentioned Assumption (4) is not satisfied, the LCC in the form of the value Z_LM71_ should be determined from the quadratic equation:

(5) $$\begin{array}{c} Z_{LM71}^{2}\times (4\times k\times \eta_{3,LM71}^{2})+Z_{LM71}\times (\eta_{1,LM71}+8\times k\times \eta_{3,LM71}\times \eta_{3,rs}-4\times k\times \eta_{3,LM71})+\\ +(\eta_{1,rs}+4\times k\times \eta_{3,rs}^{2}-4\times k\times \eta_{3,rs}+k-1)=0, \end{array}$$

where symbols η_1,rs_ and η_1,LM71_ were already defined in (2) and (3). For the other parameters, see the following three equations:

(6) $$\eta_{3,rs}=V_{rs,Ed}/V_{pl,Rd},$$

(7) $$\eta_{3,LM71}=V_{LM71,Ed}/V_{pl,Rd},$$

(8) $$k=1-M_{f,N,Rd}/M_{pl,N,Rd},$$

in which M_f,N,Rd_ represents the design value of the plastic bending resistance of a cross-section consisting of the flanges only (i.e., without the contribution of the web) and M_pl,N,Rd_ is the design value of the plastic bending resistance of the entire cross-section.

Since the value of the shear force V_Ed_ in Relation (4) is dependent on the investigated load-carrying capacity, the calculation of LCC should run in an iterative form.

Of course, the design value of shear force should be less than shear resistance. Thus, based on the above-mentioned equations and symbols defined below, the LCC of cross-section affected by pure shear can be derived from condition η_3_ ≤ 1.0 as follows:

(9) $$Z_{LM71}=(1-\eta_{3,rs})/\eta_{3,LM71}.$$

When verifying the resistance of cross-sections, it is also necessary to verify the biaxial stress state in the web. The LCC can then be derived from the next quadratic equation:

(10) $$\begin{array}{c} Z_{LM71}^{2}\times (\eta_{1,LM71}^{2}+\eta_{2,LM71}^{2}-\eta_{1,LM71}\times \eta_{2,LM71}+3\times \eta_{3,LM71}^{2})+\\ +Z_{LM71}\times (2\times \eta_{1,rs}\times \eta_{1,LM71}+2\times \eta_{2,rs}\times \eta_{2,LM71}-\eta_{1,rs}\times \eta_{2,LM71}-\eta_{2,rs}\times \eta_{1,LM71}+2,2\times \eta_{3,rs}\times \eta_{3,LM71})+\\ +(\eta_{1,rs}^{2}+\eta_{2,rs}^{2}-\eta_{1,rs}\times \eta_{2,rs}+3\times \eta_{3,rs}^{2}-1)=0, \end{array}$$

where variables η_1,rs_, η_1,LM71_, η_3,rs_, and η_3,LM71_ were already defined before, while designations η_2,rs_ and η_2,LM71_ represent the influence of local vertical stress in the web if the local vertical force is present (e.g., a sleeper on the top flange of the riveted stringer). They can be calculated on the basis of equations:

(11) $$\eta_{2,LM71}=\sigma_{z,LM71,Ed}/(f_{y}/\gamma_{M0}),$$

(12) $$\eta_{2,rs}=\sigma_{z,rs,Ed}/(f_{y}/\gamma_{M0}),$$

where σ_z,LM71,Ed_ represents the value of vertical stresses in the web due to vertical variable rail traffic load effects represented by the wheels of LM71 including dynamic factors and σ_z,rs,Ed_ is the design, combination, or group value of vertical stresses in the web due to other load effects acting simultaneously with the vertical rail traffic load.

The verification of the resistance of slender cross-sections shall respect the shear lag effects and plate buckling effects, which may be calculated by means of effective cross-sectional characteristics. More information concerning the load-carrying capacity estimation, including techniques for how LCC should be calculated in the case of compressed member buckling and/or the loss of lateral and torsional stability due to bending, can be found in [10,11].

**Figure 9:**
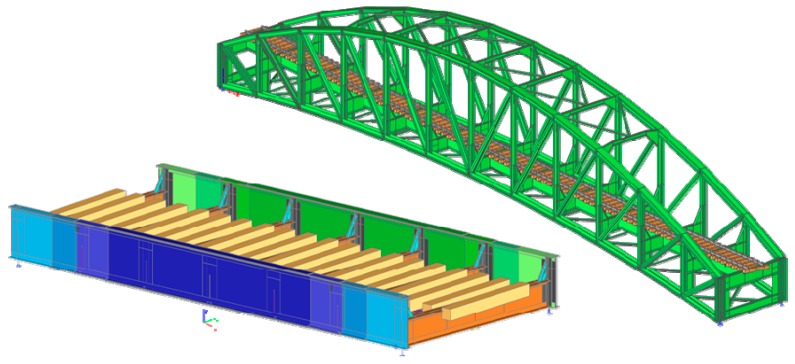
Global view at the FEM models of Bridge 1 (bottom left) and Bridge 3 (top right).

However, the process of calculating LCC has to be preceded by a very important task, which is global analysis. For the analysis of the behavior of each bridge structure, a spatial transformation numerical model was processed taking into account the real geometrical, stiffness, and material characteristics. That is why we did not put all details concerning FEM models. Two of the executed FEM models are shown in Figure 9.

The computational models were created on the basis of long-term experience with the creation of FEM models of old and newly designed bridge structures. There is lack of space in this paper for a comprehensive description of each model and applied analysis. Therefore, only the basic features of the implemented models are given. Especially for the bridge elements, the beam finite elements were used, respecting their cross-sectional characteristics, shape variability, and mutual eccentricity. The interconnections of bridge deck members (stringer-to-crossbeam) were modeled as semi-rigid joints with stiffness on the basis of executed connections. The corner stiffeners of the main girders at the crossbeams’ locations were incorporated into the models, thus helping to approximate the real rigidity of the crossbeam connection to the main girders. Increased attention was paid to this detail to obtain stiffness that was more accurate for the lateral-torsional stability analysis of the main girders. The stiffness of bridge bearings was also taken into account. Because of the better redistribution of traffic load to the stringers, the complete railway track (rails and sleepers) was included in all models so that the wheel forces from the traffic could be redistributed more correctly, but at the same time, the model of the rail track did not significantly affect the bridge deck behavior.

By utilization of the FEM, the necessary internal forces to determine the load-carrying capacities were determined by the elastic analysis. As riveted bridges are not supposed to behave in the plastic zone, the ultimate states were defined as the first occurrence of plastic strain (no matter if it was in the steel plates or in a rivet).

All relevant loads were included in the global analyses. Then, internal forces, stresses, and deformations produced by vertical variable rail traffic load were used from the design, combination, or group values caused by other load effects. After that, the process of the determination of relevant LCC could started.

## 3. Results of the Study

Baseline results of calculated actual LCC in the study are summarized in Table 2. Decreases of the cross-sectional area A, cross-section modulus W_y_, and the load-carrying capacity Z_LM71_ due to the corrosion losses are given in the table. In addition, the relative values of load-carrying capacity decrement are also presented. For determination of the load-carrying capacity values, corresponding equations from (1–12) given in previous section were utilized.

In order to specify the development of reduction of LCC over time, it would be necessary to know the corrosion rate data at a given location. Moreover, it was evident that some parts of the cross-section corroded significantly more quickly, and others were slower. It depended on the position of the element in the structure and on the shape of the element itself. Thus, one of the dominant parameters seemed to be the so-called position coefficient in the structure [18].

Partial results of LCC estimation from the bending resistance of Stringer 3 in Table 2, were already a part of the study [19].

**Figure 10:**
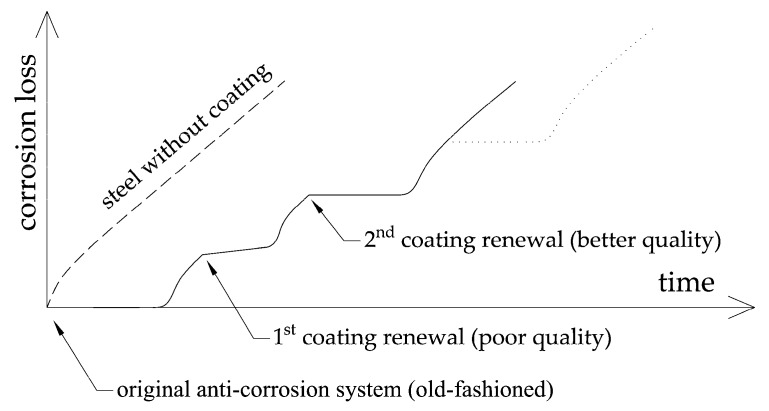
Possible time-corrosion loss relationship in the case of old bridge structure.

Other necessary data for correct time analysis and subsequent prediction of the development of corrosion and hence load-carrying capacity of the bridge were undoubtedly data about the renewals and repairs of the coating system in the past. For most of conventional bridges, this information was unobservable, and if so, there was no knowledge about the quality of the works and about the quality of the materials used nor their durability. Hence, it was evident that the time-corrosion relationship in the case of bridges had an irregular course similar to that shown in Figure 10.

Generally, it is not possible to recover the whole history of anti-corrosion adjustments and renewals on a bridge structure. Moreover, in the case of the corrosion rate at a given site, only not very accurate estimates can be made usually. In such cases, there is no possibility to reliably estimate degradation development without longer corrosion in situ measurements.

**Figure 11:**
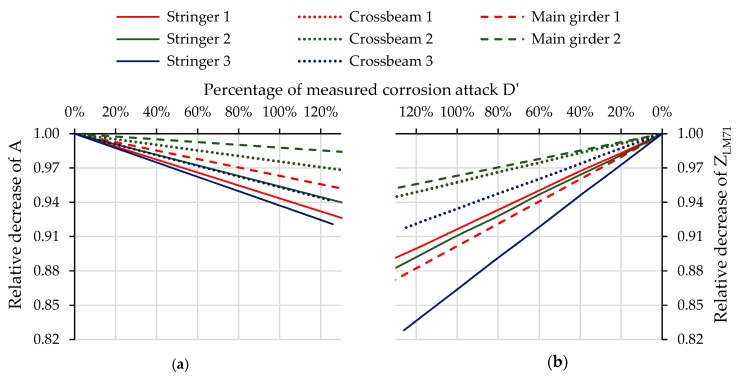
Relative decrease of the parameters of cross-sections as a function of the gradual increase of corrosion attack D’: (**a**) relative decrease of the cross-sectional area A; (**b**) relative decrease of the load-carrying capacity Z_LM71_ of the same cross-sections.

Consequently, for comparison of the reduction of LCC and its prediction in the future, the percentage values of corrosion attack D’ to the measured actual values were simply used. The right graph in Figure 11 shows the dependence of the decrease of the cross-sectional area of riveted I-sections due to degradation caused by the corrosion process. At the same time, the decrease of the load-carrying capacity Z_LM71_ of the same cross-sections is presented in the left graph.

**Figure 12:**
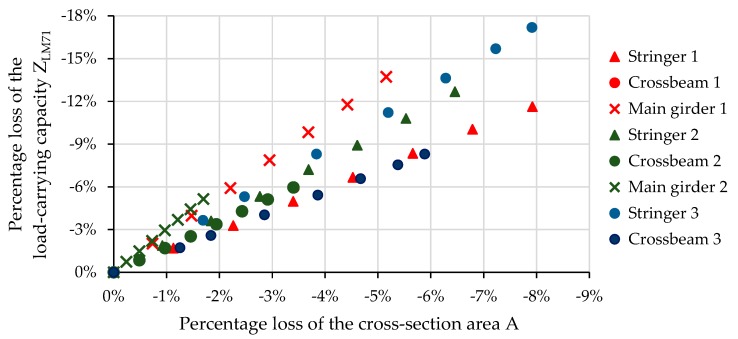
Relation between the losses of the cross-sectional area and load-carrying capacity due to corrosion.

From Figure 11, it is evident that decrease process of both the cross-sectional area A and load-carrying capacity was almost in a perfect linear relationship with corrosion attack D’. Interestingly, this conclusion applied to each of the eight cases examined in the presented study, no matter which part of the cross-section was attacked by corrosion and how big the differences in corrosion attack observed within a cross-section were. However, the decrease of the load-carrying capacity was stronger than the decrease of the corresponding cross-sectional area, which was due to the fact that the LCC of the cross-section was reduced by the other load effects. The data shown in Figure 12 indicate that the decrease in calculated load-carrying capacity was indeed faster than the decrease in cross-sectional area. From the results in the graph, it could be assumed that the percentage decrease in the load-carrying capacity at the cross-sections solved in the study was at least 1.4 times and at most 2.8 times higher compared to the loss of cross-sectional area (i.e., corrosive loss throughout the cross-section).

## 4. Conclusions and Discussion

The results of the study focus on the effect of the corrosion of steel structures on the reliability of bridge structures showed a practically linear course of decreasing cross-sectional area and load-carrying capacity of observed elements of the bridge structures due to increasing corrosion losses during their service life. A similar dependence was observed when determining the load-carrying capacity of the riveted I-section from:

- Bending resistance or combined with axial force: Equation (1)
- Combination of bending, normal force, and shear force: Equation (5)
- Shear resistance of the web: Equation (9)
- Resistance of the cross-sectional web to the biaxial stress state caused by normal stresses in the longitudinal and vertical directions in combination with the shear stresses: Equation (10)

However, the governing criteria for LCC may change over time due to corrosion losses [20]. Thus, the linear dependence can be disrupted, especially in the case of very severe damages of the cross-section due to corrosion. It seemed that the speed of reduction of load-carrying capacity could be approximately 1.5–3.0 times faster than the corrosion process speed expressed by the corrosion loss within the cross-section. The faster reduction in LCC compared to the reduction of the corresponding cross-sectional area emphasized the importance of monitoring the effect of corrosion on the static safety of the bridge structure.

Neglecting inspections can lead to substantial degradation and a consequent decrease of the load-carrying capacity [21]. Thus, only perfect up-to date protection of steel together with regular periodic inspections and basic routine maintenance can ensure the required service life and save much money. Underestimating corrosion usually results in the poor condition of bridges, requiring major repairs and reconstruction [22]. Moreover, the careful inspection activities and records from them may in the future provide valuable data for a qualified estimation of the corrosion rate for a particular bridge object or its critical elements, and thus for LCC determination and remaining service life estimation.

As already mentioned, more relevant data for the evaluation of the influence of corrosion on any steel structural bridge element can be derived only from long-term measurements. Therefore, experimental investigation on real structures or on specimens located near or directly on bridges are needed. In many European countries, these data are processed for structural steel for many years, as in [23,24,25]. In some countries, extensive research has also been devoted to weathering of steel [26,27]. Similar measurements are running also in the Slovak Republic [28,29]. The data will also be used to determine inputs for refining the corrosion map of Slovakia, as many more data are needed to improve its accuracy in regions.

## Figures and Tables

**Table 1:** Basic data and designations of the chosen bridges and their members.

Bridge Superstructure					Chosen Member		
No./type	Year/age	Span	Figure		Designation	Length	Section
Bridge **1**	1877	10.92 m	Figure 1		Stringer 1	1.82 m	Figure 4a
plate girder	142 years				Crossbeam 1	4.60 m	Figure 4b
					Main girder 1	10.92 m	Figure 4c,d
Bridge **2**	1910	22.90 m	Figure 2		Stringer 2	2.29 m	Figure 5a
plate girder	109 years				Crossbeam 2	5.24 m	Figure 5b
					Main girder 2	22.90 m	Figure 5c,d
Bridge **3**	1943	57.40 m	Figure 3		Stringer 3	4.20 m	Figure 6a
truss girder	76 years				Crossbeam 3	5.90 m	Figure 6b

**Table 2:** Results of the load-carrying capacity (LCC) of chosen members.

Element of the Bridge	Age of the Bridge	Cross-Sectional Area	Section Modulus	LCC from Bending and Axial Load: Equation (1) or (5)		LCC from Biaxial Stress State in Web: Equation (10)		LCC Derived from Pure Shear: Equation (9)	
	t (y)	A (mm^2^)	W_y_ (mm^3^)	Z_LM71_	Z_t_/Z_t0_	Z_LM71_	Z_t_/Z_t0_	Z_LM71_	Z_t_/Z_t0_
Stringer 1	0	12,300.0	1,540,127.1	0.639	1.000	1.585	1.000	–	–
	142	11,733.0	1,477,247.9	0.582	0.911	1.505	0.950	–	–
Crossbeam 1	0	17,186.0	3,348,120.4	0.668	1.000	0.958	1.000	1.661	1.000
	142	16,768.8	3,223,218.9	0.642	0.961	0.918	0.958	1.590	0.957
Main girder 1	0	33,224.0	1,3455,418.9	0.818	1.000	0.875	1.000	1.224	1.000
	142	32,821.2	1,3280,169.9	0.806	0.985	0.861	0.984	1.179	0.963
Stringer 2	0	13,056.0	1,799,326.9	0.886	1.000	1.940	1.000	–	–
	109	12,317.4	1,701,602.0	0.812	0.916	1.814	0.935	–	–
Crossbeam 2	0	19,000.0	3,997,194.4	0.465	1.000	0.767	1.000	1.490	1.000
	109	18,536.0	3,870,092.9	0.448	0.963	0.739	0.963	1.427	0.958
Main girder 2	0	57,984.0	4,1565,670.1	0.908	1.000	1.109	1.000	1.436	1.000
	109	55,848.0	4,0166,758.5	0.868	0.956	1.049	0.946	1.295	0.902
Stringer 3	0	20,878.0	3,954,636.7	1.204	1.000	1.236	1.000	–	–
	76	19,567.0	3,572,712.3	1.040	0.864	1.102	0.892	–	-
Crossbeam 3	0	31,032.0	1,0593,420.7	1.404	1.000	1.043	1.000	1.736	1.000
	76	29,582.8	1,0216,187.5	1.344	0.957	1.016	0.974	1.622	0.934

## References

[1] Leygraf C., Wallinder I.O., Tidblad J., Graedel T. (2016). Atmospheric Corrosion.

[2] Hays G.F. (2010). Now is the Time. The World Corrosion Organization, Editorial. https://www.scientific.net/AMR.95.-2.pdf.

[3] Schmitt G. (2009). Control global needs for knowledge dissemination, research, and development in materials deterioration and corrosion control. World Corrosion Organ..

[4] Wiśniewski D.F., Casas J.R., Ghosn M. (2012). Codes for safety assessment of existing bridges—Current state and further development. Struct. Eng. Int..

[5] Adey B., Bailey S., Das P., O’Brien E.J., González A. (2004). Cost345—Procedures Required for Assessing Highway Structures, European Cooperation in the Field of Scientific and Technical Research.

[6] Piau J.M. (2006). SAMARIS—Sustainable and Advanced Materials for Road Infrastructures.

[7] (2007). Sustainable Bridges—Assessment for Future Traffic Demands and Longer Lives.

[8] (2009). ARCHES—Assessment and Rehabilitation of Central European Highway Structures.

[9] (2018). UIC 778-2—Recommendations for Determining the Carrying Capacity and Fatigue Risks of Existing Metal Bridges.

[10] Vičan J., Gocál J., Hlinka R., Odrobiňák J., Moravč M., Koteš P. (2015). Určovanie zaťažiteľnosti Železničných mostov/Determination of Load Carrying Capacity of Railway Bridges.

[11] Vičan J., Gocál J., Hlinka R., Odrobiňák J., Moravčík M., Koteš P. (2015). Metodický pokyn pro určování zatížitelnosti železničních mostních objektů/Methodological Guideline for Determination of Load Carrying Capacity of Railway Bridges.

[12] Vičan J., Koteš P. (2019). Hodnotenie Existujúcich Mostných Objektov/Evaluation of Existing Bridges.

[13] EN 1991-2 (2003). Eurocode 1: Actions on Structures—Part 2: Traffic Loads on Bridges.

[14] Vičan J., Gocál J., Odrobiňák J., Koteš P. (2016). Existing steel railway bridges evaluation. Civ. Environ. Eng..

[15] Vičan J., Odrobiňák J., Koteš P. (2016). Determination of load-carrying capacity of railway steel and concrete composite bridges. Key Eng. Mater..

[16] Gomes W.J., Beck A.T., da Silva C.R. (2012). Modeling Random Corrosion Processes via Polynomial Chaos Expansion. J. Braz. Soc. Mech. Sci. Eng..

[17] Kamiński M., Szafran J. (2017). Bridges for pedestrians with random parameters using higher order Stochastic Finite Element Method. Int. J. Appl. Mech. Eng..

[18] Křivý V., Urban V., Fabian L. (2013). Experimental investigation of corrosion processes on weathering steel structures. Key Eng. Mater..

[19] Odrobiňák J., Gocál J. (2018). Experimental measurement of structural steel corrosion. Proc. Struct. Integr..

[20] Kayser J.R., Nowak A.S. (1989). Capacity loss due to corrosion in steel-girder bridges. J. Struct. Eng..

[21] Odrobiňák J., Hlinka R. (2016). Degradation of steel footbridges with neglected inspection and maintenance. Proc. Eng..

[22] Ágocs Z., Brodniansky J., Vičan J., Ziólko J. (2004). Assessment and Refurbishment of Steel Structures.

[23] Morcillo M., Simancas J., Feliu S., Kirk W., Lawson H. (1995). Long-Term Atmospheric Corrosion in Spain: Results after 13–16 Years of Exposure and Comparison with Worldwide Data. Atmospheric Corrosion.

[24] Kreislová K., Knotková D. (2017). The results of 45 years of atmospheric corrosion study in the Czech Republic. Materials.

[25] Tidblad J. (2012). Atmospheric corrosion of metals in 2010-2039 and 2070-2099. Atmos. Environ..

[26] Morcillo M., Chico B., Díaz I., Cano H., Fuente D.D.L. (2017). Atmospheric corrosion data of weathering steels. A review. Corr. Sci..

[27] Křivý V., Kubzová M., Konečný P., Kreislová K. (2019). Corrosion Processes on Weathering Steel Bridges Influenced by Deposition of De-Icing Salts. Materials.

[28] Odrobiňák J., Gocál J., Jošt J. (2017). NSS test of structural steel corrosion. Roczniki Inżynierii Budowlanej.

[29] Koteš P., Vičan J., Ivašková M. (2016). Influence of reinforcement corrosion on reliability and remaining lifetime of RC bridges. Mater. Sci. Forum.

